# Corneal Adverse Events Associated with SARS-CoV-2/COVID-19 Vaccination: A Systematic Review

**DOI:** 10.3390/vaccines11010166

**Published:** 2023-01-12

**Authors:** Lana Kuziez, Taher K. Eleiwa, Muhammad Z. Chauhan, Ahmed B. Sallam, Abdelrahman M. Elhusseiny, Hajirah N. Saeed

**Affiliations:** 1 Saint Louis University School of Medicine, St. Louis, MO 63104, USA; 2 Department of Ophthalmology, Benha University, Benha 13518, Egypt; 3 Department of Ophthalmology, Harvey and Bernice Jones Eye Institute, University of Arkansas for Medical Sciences, Little Rock, AR 72205, USA; 4 Department of Ophthalmology, Illinois Eye and Ear Infirmary, University of Illinois at Chicago, Chicago, IL 60661, USA; 5 Department of Ophthalmology, Massachusetts Eye and Ear, Harvard Medical School, Boston, MA 02114, USA; 6 Department of Ophthalmology, Loyola University Medical Center, Maywood, IL 60611, USA

**Keywords:** coronavirus, COVID-19 vaccine, corneal complications, corneal graft rejection, keratoplasty, keratitis, viral keratitis, herpes zoster, herpes simplex, vaccination

## Abstract

Vaccines against coronavirus disease 2019 (COVID-19) have played an important global role in reducing morbidity and mortality from COVID-19 infection. While the benefits of vaccination greatly outweigh the risks, adverse events do occur. Non-ocular adverse effects of the vaccines have been well-documented, but descriptions of ophthalmic effects remain limited. This systematic review aims to provide an overview of reported cases of corneal adverse events after receiving vaccination against COVID-19 and to compile existing clinical data to bring attention to these phenomena. Our review discusses corneal graft rejection, including proposed mechanisms, herpetic keratitis, and other reported corneal complications. Ophthalmologists and primary care physicians should be aware of such possible associations.

## 1. Introduction

Severe acute respiratory syndrome coronavirus 2 (SARS-CoV-2) is a highly transmissible virus that caused the coronavirus disease 2019 (COVID-19) pandemic. COVID-19 is known to have multisystem effects ranging from respiratory failure to coagulopathy [1]. More recently, ophthalmic manifestations of the infection have been identified and include conjunctivitis, scleritis, cranial nerve palsies, orbital inflammatory disease, and various posterior segment diseases [2,3,4]. The first vaccine against SARS-CoV-2 was made available in late 2020 under emergency authorization by the United States (U.S.) Food and Drug Administration. Since then, various vaccine types have been distributed globally, more than 12 billion doses have been administered, and 67% of the world population has received at least one dose. Despite copious data showing vaccination to be the most effective intervention in mitigating the spread of COVID-19, hesitancy to receive the vaccine continues both in the U.S. and globally, citing low trust in the science and the safety of vaccines [5]. As with almost any intervention, acute adverse effects do occur with SARS-CoV-2 vaccination and have largely been reported to be those common to vaccination in general, though more serious adverse effects may also be associated [6]. Organ-specific adverse events are largely limited to case series and case reports. Case reports and retrospective case series have described possible associations between the administration of the SARS-CoV-2 vaccine and the onset of ophthalmic complications involving the eyelid, orbit, cornea, retina, and other ocular structures [7,8,9,10,11,12,13,14,15]. There are numerous reports of corneal complications in particular. In this review, we aim to summarize the literature regarding corneal adverse events following SARS-CoV-2 vaccination and address concerns regarding vaccination.

## 2. Methods

We performed a systematic review of all appropriate literature guided by the Preferred Reporting Items for Systematic Reviews and Meta-Analysis Statement (PRISMA) [16]. We performed a search of all English-language literature on PubMed, searching for publications that matched the search terms “coronavirus”, “COVID-19”, “SARS-CoV-2”, or “severe acute respiratory syndrome”, and “cornea” or “corneal”, and “vaccine”.

**Figure 1:**
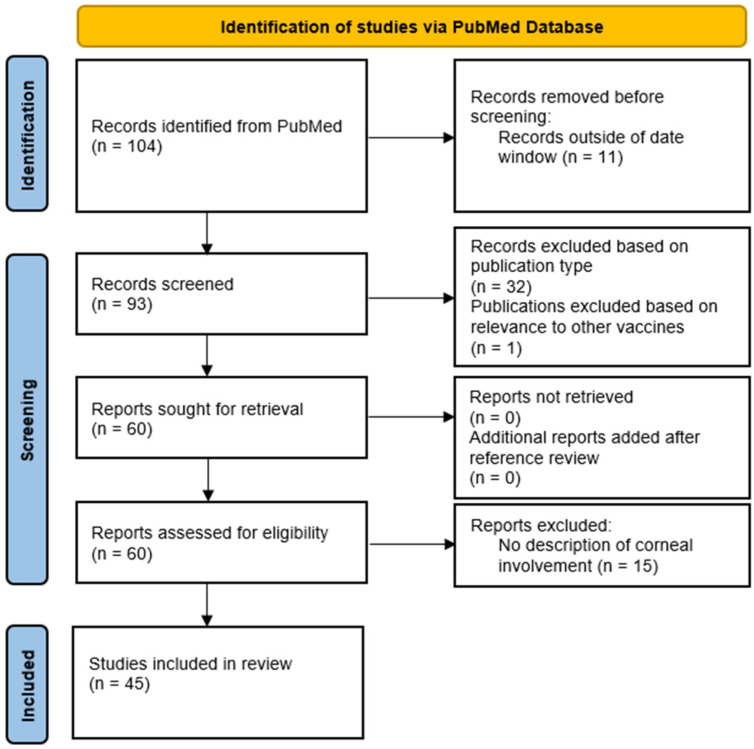
PRISMA flow diagram for this systematic review of the literature.

The search yielded case reports, case series, systematic reviews, literature reviews, and correspondences. All publications between 1 July 2021 and 1 November 2022 were included. These dates include the first described adverse effects of the SARS-CoV-2 vaccine since its first use in December 2020 to the time of this review. We included all case reports, case series, and correspondences that presented new patient data and reviewed corneal involvement of post-vaccination complications. All systematic reviews, literature reviews, meta-analyses, or replies to the author that did not present new patient data were excluded. One publication was further excluded, as it described the adverse effects of a non-SARS-CoV-2 vaccine. The references of remaining publications were reviewed for inclusion as well. The full texts of all remaining literature were screened and included if any corneal involvement was described. In total, of 93 results meeting search terms and within the desired dates, 32 were excluded based on publication type, one was further excluded due to its description of reactions to a different vaccine, and 15 were excluded due to having no described corneal involvement. In total, 45 publications met the criteria for inclusion (Figure 1). Data collected included age, sex, vaccine type, vaccine dose, the interval from vaccination to symptoms, and treatment. Where appropriate, the manifestations, corneal transplant type, time from transplant to vaccination, and corneal transplant outcomes were recorded. Continuous variables were reported using the mean, standard deviation, and range.

Cases were categorized based on the described corneal pathology. The quality of studies was evaluated using criteria published by the Task Force for Reporting Adverse Events of the International Society for Pharmacoepidemiology (ISPE) and the International Society of Pharmacovigilance (ISoP) [17]. The criteria include 12 elements: title, demographic, medical history, health status, physical examination, drug identification, dosage, administration/drug reaction interface (representing the interval), concomitant therapies, adverse event, and discussion. Publications were scored 1 point for containing an element and 0 points for the absence of an element. Therefore, publications with all criteria were given 12 points.

## 3. Results

In review, we identified 45 publications describing corneal complications associated with SARS-CoV-2 vaccination. Twenty publications described corneal graft rejection, 19 described herpetic corneal disease, and 6 case reports described unique corneal pathology. These studies included 90 eyes of 82 patients. In total, we reviewed data for 36 eyes (33 patients) with corneal graft rejection, 46 eyes (43 patients) with herpetic corneal disease, 2 eyes (1 patient) with corneal melting, 2 eyes (1 patient) with corneal edema, 2 eyes (2 patients) with limbal stem cell transplant rejection, 1 eye with peripheral ulcerative keratitis, and 1 eye with marginal keratitis. Information for each case of corneal graft rejection and herpetic keratitis are presented in Table 1 and Table 2, respectively. Of note, herpetic disease often refers to herpes simplex or herpes zoster. However, one case, as described later, discusses a case with suspected infection from cytomegalovirus, HSV, or VZV, all part of the herpesvirus family; viral keratitis of another etiology has not been reported in this population. As such, we have referred to all instances of viral keratitis in this review as herpetic keratitis. Furthermore, cases of keratitis associated with herpes simplex are referred to as herpes simplex keratitis (HSK), while cases associated with herpes zoster are referred to as herpes zoster keratitis (HZK); however, HZK with cutaneous V1 dermatomal involvement is referred to as herpes zoster ophthalmicus (HZO).

Patients received mRNA, viral vector, or inactivated vaccines, including the CoronaVac (Sinovac Biotech Ltd., Beijing, China), BBIBP-CorV (Sinopharm, Beijing, China), AZD1222 (AstraZeneca, Cambridge, UK), mRNA-1273 (ModernaTX, Inc., Cambridge, MA, USA), and BNT162b2 (BioNTech, Mainz, Germany) vaccines.

The mean quality score of the 45 publications was 11.4 out of 12 possible points. Twenty-five scored 12 points, fourteen scored 11, five scored 10, and one scored 9 (Table 3). Indication of current health status and concomitant therapies were the most commonly missed elements.

## 4. Corneal Graft Rejection

Of the 45 publications we reviewed, 20 described acute corneal graft rejection in 36 eyes of 33 patients. Of the 33 patients, 16 were male, and 17 were female. The mean age was 60.1 ± 16.3 years (median: 63.5; range: 15–94). Acute rejection occurred following the patient’s first dose of the vaccine in 26 (72%) eyes and after the patient’s second dose in 10 (28%) eyes. There were no reports of rejection after any booster doses. Among all patients, 1 (1 eye, 3%) received the CoronaVac vaccine, 2 (2 eyes, 6%) received the BBIBP-CorV vaccine, 9 (9 eyes, 25%) received the AZD1222 vaccine, 7 (7 eyes, 19%) received the mRNA-1273 vaccine, and 14 (17 eyes, 37%) received the BNT162b2 vaccine. The time from the most recent vaccine to presentation was 11.2 ± 0.4 days (median: 8.5; range: 1–42). Cases were mostly represented by unilateral rejection in the setting of a single graft; however, there were three cases of bilateral corneal graft rejection.

Various keratoplasty techniques were reported, including penetrating keratoplasty (PKP), Descemet’s stripping (automated) endothelial keratoplasty (DSAEK), Descemet’s membrane endothelial keratoplasty (DMEK), and femtosecond laser-assisted endothelial keratoplasty (FLEK). After reviewing the most recent keratoplasty procedure among the 36 eyes, we found 20 eyes (55.6%) to have undergone a PKP, 1 eye (2.8%) a FLEK, 8 eyes (22.2%) a DMEK, and 7 eyes (19.4%) a DSAEK. The time from the most recent keratoplasty to the reported rejection episode was a mean of 6.2 years and ranged from 14 days to 25 years with a median of 3.5 years. Notably, the standard deviation of the interval time data was greater than the mean, indicating a high variation in the data.

The outcomes of the rejection episodes were mixed. Twenty-two eyes (36%) had resolution, 7 eyes (19%) had improvement, and 6 eyes (17%) had no improvement. The outcome was not reported for 1 eye. Of the 20 eyes that underwent PKP, 14 (70%) had resolution, 2 (10%) had improvement with no further descriptions of the disease course, and 3 (15%) had no improvement (1 outcome was unreported). Of the 8 eyes that underwent DMEK, 2 (25%) had resolution, 4 (50%) had improvement with no further descriptions of disease course, and 2 (25%) had no improvement. Seven eyes underwent DSAEK, and 5 (71%) had resolution; improvement was seen in 1 eye (14%) with no further descriptions of the disease course, and no improvement was seen in 1 eye (14%). Resolution was reported for the one eye that underwent FLEK. Analysis of outcomes by vaccine dose revealed a greater incidence of resolution in patients who had rejection after the first dose versus the second. Among 26 eyes that had rejection after the first vaccine, 18 (69%) had resolution, 3 (12%) had improvement, and 4 (15%) had no improvement; among 10 eyes with rejection after their second dose, 5 (50%) eyes had resolution, 4 (40%) had improvement, and 1 (10%) had no improvement. Comparisons of outcomes by vaccine type demonstrated a similar incidence of resolution in the groups that received the AZD1222 vaccine (7 of 9 eyes, 78%) and the BNT162b2 vaccine (13 of 17 eyes, 76%). The incidence of resolution was lower for the groups that received the BBIBP-CorV (1 of 2 eyes, 50%), mRNA-1273 (1 of 7 eyes, 14%), and the CoronaVac (0 of 1 eye, 0%) vaccines.

Molero-Senosiain et al. reported on five patients who presented symptoms within 1 month of vaccination. One case involved a 72-year-old female with a history of Fuchs endothelial dystrophy (FED) requiring three DSAEKs in her right eye, and three PKPs, and one DSAEK in her left eye, with the most recent surgeries being a DSAEK in the right eye and a PKP in the left, five and eight years prior to presentation, respectively. The authors did not report any underlying risk factors for the multiple graft failures that the patient had [25]. Fourteen days after her first dose of the BNT162b2 vaccine, she presented with blurred vision and a visual acuity (VA) of 20/80 in the right eye. Prior to this episode, she had poor VA in her left eye (counting fingers) due to a failed PKP and a VA of 20/50 in her right eye. Examination revealed graft edema in only the right eye. There were no keratic precipitates (KPs) or anterior chamber reaction. Resolution was achieved by increasing the frequency of her pre-rejection maintenance treatment of topical dexamethasone, and her final VA was 20/60. The second case was an 82-year-old woman with a similar history of FED requiring DSAEK of the right eye four years prior. She presented 14 days following the BNT162b2 vaccine with blurred vision and a reduction in VA from her baseline of 20/40 to 20/100. On exam, she had an edematous graft without KPs and no anterior chamber reaction. Resolution was achieved with topical steroids, and VA improved to 20/60. The remaining three cases in this series involved a 55-year-old male, 61-year-old male, and 48-year-old female, all with a history of keratoconus that was treated with PKP and with pre-rejection VAs of 20/66, 20/100, and 20/45 which worsened to counting fingers, 20/350, and counting fingers, respectively. They all reported blurred vision following the administration of the AZD1222 vaccine. Although all eyes presented with corneal edema, two eyes also had Descemet’s membrane folds, one had KPs, and one had an anterior chamber reaction. Treatment for all patients involved topical steroids, although one patient needed intravenous (IV) methylprednisolone after a poor response to topical steroids. All patients achieved reversal of rejection and improvement in vision (20/100 for the female patient and 20/125 for the others).

Similar cases of acute rejection have been reported by Shah et al. in patients receiving the mRNA-1273 vaccine. In their case series, they describe four patients who received the vaccine and developed signs of rejection within two weeks of administration [34]. A 74-year-old male who had a DMEK for pseudophakic bullous keratopathy three months prior and was compliant with once-daily topical fluorometholone reported blurred vision following his first vaccination dose. The exam was significant for a VA of 20/60 from a baseline of 20/25 an endothelial rejection line, KPs, Descemet folds, and corneal edema with a central corneal thickness (CCT) of 743 µm. Within two days of every-2-h prednisolone use, the CCT decreased to 705 µm. He had his second vaccine dose while continuing topical prednisolone and did not experience further complications; after several weeks, his vision improved to 20/40, and CCT further decreased to 655 µm. The authors described another case of a 69-year-old woman with FED requiring bilateral DSAEK 6 years prior and with a pre-rejection VA of 20/25. She presented with a worsening vision in her left eye 14 days after her second dose of the mRNA-1273 vaccine. Slit lamp exam showed conjunctival injection, anterior chamber reaction, and stromal edema (CCT 719 µm) of the left eye; the right eye examination was unremarkable other than the presence of an intact DSAEK graft. Within 3 weeks of topical difluprednate initiation, her VA improved from 20/50 at presentation to 20/30, and there was a resolution of the anterior chamber cell and improvement of stromal edema (CCT 633 μm). The remaining two cases described in this series included a 61-year-old female and a 77-year-old male with a history of a PKP 2.5 and 22 years prior, respectively, and baseline VA of 20/40 and 20/25, respectively. Both patients reported declining vision 1 week after their second dose of the mRNA-1273 vaccine. The exam revealed VA of 20/80 and 20/60 in the affected eyes, respectively, and corneal graft edema, conjunctival inflammation, and KPs in both patients. An endothelial rejection line was also seen in one patient and an anterior chamber reaction in the other. Both patients received topical prednisolone for several weeks with an improvement of VA to 20/60 and 20/40, respectively, and resolution of graft edema, KPs, and conjunctival inflammation. In this series, Shah et al. presented four cases that suggest a temporal association between the mRNA SARS-CoV-2 vaccine and acute corneal graft rejection while recognizing the need for wider population-based and comparative studies to investigate the incidence of graft rejection and any association with SARS-CoV-2 vaccination.

In their case series, Balidis et al. described four cases that responded variably to treatment [19]. They reported on a 77-year-old woman who underwent a DMEK for pseudophakic bullous keratopathy 20 months prior and had three episodes of graft rejection within eight months of transplantation; two episodes were associated with HSK. The third episode required escalation to systemic steroid therapy and coverage for herpetic keratitis; ultimately, the graft failed. After regrafting, the patient continued topical corticosteroids and oral antivirals. At her 12-month appointment post-regraft, her graft was noted to be the clearest since the transplant was performed. Soon after, she received the mRNA-1273 vaccine and noticed blurred vision 7 days later; her exam revealed KPs and corneal edema. Subconjunctival dexamethasone, topical corticosteroids, and hypertonic eye drops did not improve the rejection episode, and she required IV dexamethasone. Improvement was noted at the four-week mark. The authors also reported on a 64-year-old woman with a history of PKP for keratoconus 2 years prior with no complications and a CCT of 470 µm at baseline. One week after the patient’s second dose of the mRNA-1273 vaccine, she experienced blurred vision. Anterior chamber reaction and stromal edema (CCT 585 µm) were noted on slit-lamp examination. Topical and intracameral dexamethasone did not achieve resolution, and the edema persisted after four weeks of therapy; the authors did not report any additional interventions. Patient three in this series was a 69-year-old male who underwent PKP of the right eye 22 months prior for post-herpetic corneal scarring. He received the first dose of the AZD1222 vaccine and experienced reduced vision five days later in that eye (VAs not reported). The exam revealed KPs and corneal edema (CCT 757 µm). Treatment involved subconjunctival dexamethasone injections, oral methylprednisolone, and topical dexamethasone. Stromal edema initially did not improve but did so after 8 weeks of treatment (CCT 660 µm). Finally, the authors presented a 63-year-old male with a history of DSAEK of the left eye for FED and repeated DSAEK 9 months prior to presentation due to graft failure. His blurred vision began 10 days after the first dose of the AZD1222 vaccine, with VA reduced to counting fingers at one meter from 20/40 at baseline. Again, corneal edema and KPs were seen. Treatment with topical dexamethasone was begun, but the edema persisted at the 3-week follow-up visit. The authors did not report any further follow-up.

Similar reports of acute graft rejection in patients with various keratoplasty types, variable times since procedure, and repeat graft histories have been made [18,20,26,27,31,33,35,36,37]. In all cases that reported treatment, patients were treated with topical steroids; in some cases, oral steroids, intracameral dexamethasone, subconjunctival dexamethasone, or IV steroids were used concurrently or subsequently. Despite undergoing prescribed treatment, five total patients reported by Yu et al., Simão et al., Forshaw et al., and Balidis et al. ultimately had graft failure following SARS-CoV-2 vaccination [19,22,35,37]. Yu et al. described a 51-year-old male patient who had acute rejection 3 weeks post-PKP [37]. He was receiving topical steroids but ultimately had graft failure and progression of glaucoma in the setting of increased steroid use. Simão et al. presented a patient who had acute PKP graft rejection after vaccination which improved with topical dexamethasone. After her second dose of the CoronaVac vaccine, she returned with the same clinical presentation; the same treatment was repeated with no recovery, and ultimately had graft failure. Lastly, Forshaw et al. reported on a 94-year-old female with bilateral DMEK rejection 14 days after receiving a SARS-CoV-2 vaccine (unspecified type) [22]. Treatment with topical dexamethasone/tobramycin and hypertonic saline failed to improve her corneal edema. She received a repeat DMEK in both eyes with an overall improved corneal clarity. The remaining two cases were described by Balidis et al. as above.

### Discussion

Corneal transplantation is among the most common and successful solid organ transplantations [63]. While rejection is understood to be rare following vaccination in general, the phenomenon is likely underreported [45,64]. A survey of cornea specialists in 2021 revealed at least 34 anecdotal keratoplasty rejection episodes related to vaccines, but that same study also noted that only four articles describing a total of 12 cases of an association between recent vaccination and corneal transplant rejection had been published over 30 years [65]. High-quality studies on the association between vaccines and corneal graft rejection have not been done, and the evidence is thus far inconclusive. While no formal pathophysiologic link has been made between the two, a review of post-vaccination corneal graft rejection revealed associations between rejection and influenza, tetanus, hepatitis B, yellow fever, recombinant zoster, and SARS-CoV-2 vaccines [64].

Our literature review revealed 33 cases (36 eyes) of corneal graft rejection after SARS-CoV-2 vaccination, more reported cases than with any other vaccine. There are many biases that may account for this difference, but it is a difference that has not been prospectively explored and one that needs further study. On the other hand, Busin et al. examined 77 diagnosed cases of rejection among 2062 patients that received corneal transplants between January 2018 and December 2021 [66]. They found no notable increase in the number of eyes with graft rejection in 2021 when vaccination was widely implemented. In further analysis, the authors compared the incidence of graft rejection in the “risk period” (the first 60 days after vaccination) versus the incidence outside of the risk period. They found no significant increase in the incidence of rejection within the risk period. Additionally, they analyzed rejection data for patients who received a cornea transplant before receiving their SARS-CoV-2 vaccine and those who received a transplant after the vaccine and again found no significant differences. Similarly, a multi-country study by Roberts et al. found no increase in the number of corneal graft rejection cases diagnosed per month after vaccination programs were widely implemented versus in the periods prior to lockdowns and during lockdowns [67]. The data presented by both Busin et al. and Roberts et al. do not support an association between SARS-CoV-2 vaccination and corneal graft rejection. However, all published data together provide insight into the need for analysis on a greater scale to establish vaccine safety in patients with corneal grafts.

Based on the limited dataset in this review, it appears that rejection was less likely to fully resolve after DMEK as compared to PKP or DSAEK. This is an association that warrants further study, but may be due to the often lower dosage and potency of topical steroid regimen post-DMEK as compared to other forms of corneal transplantation. Clinicians should be aware of a possible association between SARS-CoV-2 vaccination and corneal graft rejection, report associations when found and take this into consideration in clinical management. It is important to note the high degree of reversibility of corneal graft rejection with appropriate management and re-emphasize the benefit of SARS-CoV-2 vaccination for individuals and communities. Clinicians may consider increasing topical steroid use in the peri-vaccination period as a relatively low-risk potentially prophylactic measure to mitigate graft rejection.

The cornea is among the few tissues in the body that have immune privilege. The unique avascular anatomy as well as the absence of lymphatic tissue within the cornea prevent access by the immune system. Additionally, the corneal layers express low amounts of major histocompatibility complexes (MHC) I and II, limiting the immune response against antigens. Regulatory T cells (Tregs) have an important role in downregulation of immune responses in the cornea. Expression of surface molecules on these cells, including cytotoxic T lymphocyte antigen-4, programmed cell death ligand-1, and forkhead box protein 3 (Foxp3), as well as secretion of interleukin-10 and transforming growth factor-β work to suppress immune activation by inhibiting activities of antigen presenting cells and CD4+ T cells and inhibiting interferon gamma production [63,68,69]. While dendritic cells exist in the central and peripheral cornea, they are suppressed by interleukin-1 receptor antagonist expressed in the cornea, further reinforcing the cornea’s exclusion from immune surveillance [68]. These mechanisms and others promote survival of corneal allografts. It has been hypothesized that the immune system activation and dysregulation occurring after vaccination may threaten these barriers and expose the corneal graft and foreign antigens to the immune system, mediating rejection [70].

Cross-reactivity between the SARS-CoV-2 antigen and MHC-antigen complexes has been proposed as a mechanism for acute rejection following SARS-CoV-2 vaccination. The BNT162b2 and the mRNA-1723 vaccines are lipid-encapsulated mRNA molecules encoding the spike protein, which is the target antigen for the humoral immune response. After vaccination, anti-spike protein titers are elevated. At this time, antibodies that are cross-reactive with corneal graft donor molecules may produce an immune response, thereby mediating rejection [30]. Another proposed mechanism is based on observed corneal responses during states of inflammatory stress. In response to stress, MHC class II and co-stimulatory molecule expression is induced in corneal epithelial cells and dendritic cells. Such inflammatory stress may be induced after vaccination and lead to allosensitization by presentation of donor antigens [68]. Similarly, inflammation within the host bed has been demonstrated to decrease the expression of Foxp3 in Tregs and disrupt differentiation of Tregs, potentially weakening the multiple mechanisms for immune modulation by Tregs [71]. Furthermore, SARS-CoV-2 vaccines elicit strong humoral and cellular immune responses, as seen with other vaccines, including a Th1-biased CD4+ response. CD4+ Th1 cells are mediators of corneal graft rejection and may play a role here [32,72].

Another possible mechanism may include an immune reaction to vaccination adjuvants, which are used to enhance the body’s immune response and lower the frequency and amount of vaccine needed to obtain adequate preventive immunity [49].

## 5. Herpetic Keratitis

Of the literature included in this review, 19 publications described the occurrence of herpetic keratitis after SARS-CoV-2 vaccination in 46 eyes of 43 patients. Twenty-six patients (60.4%) were male. Mean age was 55.7 ± 21.3 years (median: 55; range: 18–95). One patient (1 eye, 2%) received the mRNA1273 vaccine, 1 patient (1 eye, 2%) received the BBIBP-CorV vaccine, 22 patients (22 eyes, 48%) received the BNT162b2 vaccine, 4 patients (5 eyes, 11%) received the CoronaVac vaccine, and 12 patients (13 eyes, 28%) received the AZD1222 vaccine. Vaccine type was not recorded for 3 patients (4 eyes, 9%). Symptoms began an average of 9.5 ± 8.4 days (median 7; range 1–28) after vaccination in the 41 patients for which this interval was reported. Vaccination to symptom interval was not reported for 2 patients (2 eyes). Twenty-one patients (22 eyes, 49%) reported symptoms after their first vaccine, 17 patients (19 eyes, 33%) had symptoms exclusively after their second vaccine, and 2 patients (2 eyes, 5%) had symptoms after their third vaccination/booster. Three patients (4 eyes, 9%) had symptom recurrence after receiving a subsequent vaccine dose. Vaccine dose number was unreported for 3 patients. Fourteen patients (15 eyes, 33%) were diagnosed with herpes simplex keratitis (HSK) while 16 patients (16 eyes, 35%) were diagnosed with herpes zoster keratitis (HZK/HZO). Six studies involving 15 eyes of 13 patients did not specify which type of herpes virus infection was diagnosed, although based on history and exam it is likely that two of these eyes were HSK and two were HZK. Additionally, 25 patients (25 eyes, 54%) had a known history of herpetic keratitis prior to presentation for acute reactivation or recurrence of disease following vaccination.

All cases of herpetic keratitis (HK) with reported outcomes had improvement or resolution. Of the 41 eyes with reported outcomes, 24 (59%) had resolution and 17 (41%) had improvement. Of the 13 eyes with HSK and reported outcomes, 7 (54%) had resolution and 6 (46%) had improvement. Among 16 eyes diagnosed with HZK/HZO, 9 eyes (56%) had resolution and 7 (44%) had improvement. In the group diagnosed with HK after the first vaccine dose (22 eyes), 15 (68%) had resolution and 7 (32%) had improvement. In the group with HK after the second vaccine dose (14 eyes), 9 eyes (64%) had improvement and 5 eyes (36%) had resolution. Resolution was seen in the 2 eyes with HK after the third vaccine dose (100%). Among the 22 eyes with HK after receiving the BNT162b2 vaccine, 11 (52%) had resolution and 10 (48%) had improvement (outcome was unreported for 1 eye). Of the 13 eyes that had HK after receiving the AZD1222 vaccine, 8 (67%) had resolution and 4 (33%) had improvement (outcome was unreported for 1 eye). Five eyes had HK after receiving the CoronaVac vaccine and 2 (40%) had resolution while 3 (60%) had improvement. One eye with HK after receiving the BBIBP-CorV vaccine had resolution after treatment.

Al-Dwairi et al. described a case of a 50-year-old man with a history of PKP of the left eye for corneal opacity from a previous episode of herpetic keratitis years prior [38]. He received pre- and post-operative subconjunctival anti-vascular endothelial growth factor injections and had been on oral acyclovir and topical prednisolone for over a year until 7 months before his presentation. He presented almost two years post-operatively with redness, tearing, and pain in his left eye following vaccination one week prior with the BNT162b2 vaccine. The examination was significant for multiple dendritic ulcers in the graft with ciliary injection and anterior chamber cells consistent with recurrence of HSK. He ultimately had resolution of the ulcers after topical acyclovir and moxifloxacin as well as lubricant eye drops for 2 weeks. Li et al. presented a similar case of a 60-year-old woman with a history of PKP for corneal scarring due to HSK [45]. She had no episodes of acute rejection or keratitis until two days after receiving the inactivated CoronaVac vaccine. Characteristic dendritic ulcers were seen on slit lamp examination. Treatment with topical ganciclovir and discontinuation of topical steroids resolved the recurrent HSK in two weeks. The patient went on to receive her second dose of the vaccine while using topical ganciclovir without any issues.

Several cases of HZK after SARS-CoV-2 vaccination have also been reported. You et al. presented a unique case of VZV reactivation and meningitis in a 74-year-old male who initially presented with headache, forehead pain, left eyelid swelling, and photophobia [56]. He had had the second dose of the BNT162b2 vaccine 5 days prior. On exam, skin lesions involved the ophthalmic distribution of the trigeminal nerve. Ocular exam revealed pseudodendrites of the cornea, conjunctival chemosis, and hyperemia. Further investigation of the headache involved cerebrospinal fluid studies which were indicative of meningitis due to VZV. He received IV acyclovir, topical acyclovir, and topical levofloxacin. He had improvement within 3 days. Twenty days after discharge, he returned with a central epithelial defect of the same eye. Treatment was initiated with a bandage contact lens and a topical solution of ofloxacin and recombinant human epithelial growth factor. Over two months, the epithelium healed and at 6 months, exam findings were stable with some stromal haze.

Shan et al. presented a case of a 19-year-old man with no ophthalmic history who developed a suspected herpetic keratitis after both the second and third dose of the CoronaVac vaccine [54]. He initially presented three weeks after his second dose with blurred vision in both eyes and was treated with topical ganciclovir and levofloxacin but had no relief. He was later admitted for worsening symptoms and exam findings of conjunctival hyperemia, irregular corneal epithelium, and patchy corneal infiltrates. He received IV ganciclovir, topical ganciclovir, and topical cyclosporine. Within 1 week, his symptoms resolved, and exam findings and vision were improved. He received the third dose of the CoronaVac vaccine 18 months later with a recurrence of the same symptoms; exam findings were consistent with those of the previous episode. He again received topical ganciclovir and topical cyclosporine. He also received oral acyclovir and ganciclovir. At the follow-up one week later, his exam had improved, symptoms resolved, and VA returned to 20/20. Unique to this case is the recurrence of corneal disease with repeat vaccination suggesting an association between the vaccine and viral reactivation in the cornea.

Of the 14 patients with HSV reactivation, 12 had a previous history of HSK. Of the two cases with no previous HSK history, one developed into recurrent HSK after its manifestation after both the first and second doses of the vaccine. In their case series, Rallis et al. included the case of a 59-year old-male with no history of herpetic ocular disease who developed bilateral keratitis 4 days after receiving the AZD1222 vaccine [50]. Exam revealed right sided corneal dendrites and a geographic ulcer of the left eye. He received topical ganciclovir and topical corticosteroids, and oral acyclovir. Complete resolution was noted after two weeks. After his second dose of the AZD1222 vaccine, he had recurrence of herpetic keratitis. No further treatment or follow-up was reported. In contrast, of the 16 patients with HZK, only 6 had a previous history of HZK or HZO. Patients with no history of herpetic keratitis had a mean age of 56 ± 22.8 years (median 57; range 19–95); 28% were female, and 72% were male. Among patients with recurrent herpetic keratitis, the mean age was 55.6 ± 20.6 years (median 53; range 18–89); 48% were female and 52% were male. Other cases of reactivation and recurrence of HSV and VZV as described in Table 2, involved typical presentations of such infections. Treatment for herpetic keratitis included topical antivirals, oral antivirals, or a combination of both with or without additional agents.

Interestingly, a recent study using the Centers for Disease Control and Prevention Vaccine Adverse Events Reporting System demonstrated a temporal association between vaccine-associated uveitis (VAU) and SARS-CoV-2 vaccination. Among 491 patients diagnosed with VAU, 249 (20.76%) had herpes ophthalmicus (unspecified) [9]. They also noted that the benefits of vaccination outweigh the risks of VAU but that physicians should be aware of this association.

### Discussion

The reported adverse effects of the SARS-CoV-2 vaccines have included the reactivation of viral diseases, particularly herpes simplex and herpes zoster. In addition to other cutaneous reactions, McMahon et al. described 4 cases of herpes simplex flares and 10 cases of herpes zoster from among 414 reported cutaneous effects of the SARS-CoV-2 vaccine within an international database [73]. Ozdemir et al. similarly reported herpes zoster in two healthy young patients following vaccination [74]. Additional literature presents similar cases of reactivation of latent herpetic infection after vaccination [75]. In their review of the Vaccine Adverse Events Reporting System, Gringeri et al. identified 5934 cases of herpes zoster and 273 of herpes simplex infection after receiving the BNT162b2 vaccine [76]. These cases included 60 (1.01%) cases of “ophthalmic herpes zoster” and 6 (2.20%) cases of “ophthalmic herpes simplex”. They found a reporting odds ratio of 1.49 for herpes zoster and 1.51 for herpes simplex. Barda et al. found a similar risk ratio for herpes zoster of 1.43, but an unremarkable risk ratio for herpes simplex infection for the same vaccine [77]. However, Shasha et al. found a risk ratio for herpes zoster that was inconclusive for the same vaccine [78]. Conflicting evidence in the literature regarding the SARS-CoV-2 vaccines and their potential effects on latent viral disease reveals the need for further research.

In herpetic infections of the trigeminal distribution in general, the virus resides in the trigeminal ganglion after primary infection. Reactivation along the ophthalmic branch can cause corneal disease. Little is known about the potential pathogenesis of herpetic reactivation after SARS-CoV-2 vaccines, and it is unclear what role the vaccines may have on recurrent versus new-onset herpetic keratitis. Known triggers of herpes virus reactivation are induced in severe COVID-19 infections, including fever and physical stress and may be implicated in the case of vaccination [79]. It has been hypothesized that lymphopenia and lymphocyte exhaustion during the course of a severe COVID-19 infection may also contribute to reactivation [80,81]. Additionally, in the post-vaccination state, stimulation of the immune system is induced, involving increases in T-helper type 1 CD4+ and CD8+ cells. This stimulation in cellular immunity may cause a shift of naïve CD8+ cells, overwhelming the ability of virus-specific CD8+ cells to control the latent virus [82]. An additional hypothesis describes the response of toll-like receptor (TLR) signaling to vaccination against COVID-19. TLRs are known to be involved in the reactivation process of herpes viruses and serve to maintain the latency of the virus in the host. Vaccination also stimulates the release of inflammatory cytokines, which induce T and B cell immune responses but disturb antigen expression, lowering the threshold for reactivation [80,83].

## 6. Others

A single case of corneal melt after SARS-CoV-2 vaccination was reported in the literature by Khan et al. [60]. They presented the case of a 48-year-old man with a two-week history of blurred vision, photophobia, pain, and watering in both eyes. He had received the first dose of the AZD1222 vaccine 3 weeks before the start of his symptoms. On exam, he had conjunctival and ciliary congestion, with corneal melt, uveal tissue prolapse, and bilateral massive choroidal detachments found on B-scan ultrasonography. Workup for underlying autoimmune or infectious etiologies was negative. Ultimately, the patient required PKP in both eyes.

A case of bilateral corneal edema was reported by Lee and Han [61]. A 55-year-old female with a history of uneventful cataract surgery two months prior presented with sudden visual disturbance and ocular pain six days after receiving the AZD1222 vaccine. Her vision at presentation had worsened from 20/20 at baseline to 20/30 bilaterally. Slit-lamp examination revealed mild bilateral corneal edema with a CCT of the right and left eyes of 580 µm and 594 µm, respectively. Endothelial cell density was 2849/mm^2^ in the right eye and 2778/mm^2^ in the left eye. After two weeks of topical prednisolone use, her vision improved to 20/25 bilaterally, and the corneal edema had resolved in the right eye with minimal residual edema of the left eye; CCTs improved to 553 µm and 579 µm of the right and left eyes, respectively.

Marginal keratitis as an adverse effect was reported in one case by Farrell et al. [58]. A 66-year-old female presented with worsening right eye pain and redness 2.5 weeks after receiving the first dose of the mRNA-1273 vaccine. She self-medicated with antibiotic eye drops from symptom onset with no improvement. On exam, the right eye had a significant conjunctival injection, infiltrates of the cornea, and peripheral corneal vascularization. There were no epithelial defects, anterior chamber abnormalities, or discharge. The left eye was unremarkable. She was diagnosed with marginal keratitis of the right eye and started topical antibiotics and corticosteroids and had improvement within several days.

Gouveau et al. and de la Presa et al. have reported on patients experiencing rejection of limbal allografts following SARS-CoV-2 vaccination. The first by Gouveau et al. described a 72-year-old male with a history of chemical injury and subsequent keratolimbal allograft of the right eye (KLAL) 6.5 years prior; he had an uneventful post-operative course and later had a PKP and cataract extraction/intraocular lens implantation 15 months and 3.5 years after the KLAL, respectively [59]. He tolerated his medication regimen of oral tacrolimus, topical prednisolone, dorzolamide-timolol, and cyclosporine well His tacrolimus dosage was eventually decreased, and his subsequent lab work showed subtherapeutic tacrolimus levels. On routine follow-up 1 month after receiving the second dose of the BNT162b2 vaccine, he was noted to have chemosis as well as perilimbal engorgement and tortuous vessels. However, there was no corneal edema, KPs, anterior chamber reaction, or epithelial rejection line. The patient declined oral steroids and was started on topical difluprednate and tacrolimus, and his oral tacrolimus dosage was increased. Within 4 months, the KLAL segments showed improvement. In their similar case study, de la Presa et al. presented a 27-year-old with a history of limbal-stem cell deficiency secondary to contact lens use and subsequent living-related conjunctival limbal allograft (LR-CLAL) of her right eye approximately 4.5 years prior [57]. She was maintained on mycophenolate mofetil (MMF) and topical prednisolone for years with no noted complications at her regular follow-up appointments. However, 15 days after receiving the first dose of the mRNA-1273 vaccine, she presented with redness and irritation in her right eye. Exam revealed conjunctival hyperemia sparing the LR-CLAL graft and an epithelial rejection line along the limbus with no corneal infiltrates or edema. She was started on topical difluprednate, and oral prednisone, and her MMF was increased. Her symptoms improved over several days, and by two weeks, improvements were seen on the exam (fading epithelial rejection line and resolution of hyperemia). She received the second dose of the vaccine while still on increased topical and systemic immunosuppression with no complications.

Lastly, Penbe described a single case of peripheral ulcerative keratitis in a 76-year-old man with a history of PKP of the left eye 10 years prior who presented with right eye pain and blurred vision two weeks after receiving the CoronaVac vaccine [62]. On presentation, he had peripheral stromal infiltration spanning 180 degrees, corneal necrosis from the temporal limbus to the visual axis, and nodular scleritis of the right eye. The left eye had no acute changes but was opaque from the previous pathology. Work-up for autoimmune etiology was negative. He was empirically treated with IV methylprednisolone and topical moxifloxacin, dexamethasone, cyclosporine, cyclopentolate, flurbiprofen, and autologous serum drops. He was later transitioned to oral prednisone, azathioprine, and doxycycline. He received several amniotic membrane grafts over 5 weeks, and the corneal defect resolved. However, his VA had improved minimally from finger counting at 10 cm at presentation to finger counting at one meter. In the setting of an opaque left corneal graft, the decision to pursue PKP was ultimately made to restore vision to 20/100. In the postoperative follow-up 4 weeks later, the right eye corneal graft was clear.

## 7. Conclusions

Since efforts directed toward vaccinating the global population against COVID-19 began in 2021, cases of ophthalmologic adverse events occurring in the post-vaccination period have been reported. In this review, we presented cases of corneal complications following SARS-CoV-2 vaccination and discussed mechanisms theorized to be involved in acute corneal graft rejection and herpetic keratitis post-vaccination. Despite the 66 cases reviewed here, a causal relationship between the two events cannot be definitively established; more data are needed to better understand the potential interactions of the vaccine with the cornea and its effect on immune response. More data are also needed to make any correlations between ocular outcomes after COVID-19-associated corneal graft rejection and herpetic keratitis, and the variables described by the ISPE and the ISoP, including medical and medication history. Regardless, the benefits of vaccination appear to outweigh the risks and in the absence of new evidence suggesting otherwise, ophthalmologists should continue to recommend vaccination against COVID-19 for patients. At the same time, patients who have a history of corneal transplantation or herpetic keratitis should be closely monitored after vaccination and should be counseled on the signs and symptoms of graft rejection and herpetic reactivation, respectively.

## Figures and Tables

**Table 1:** Patient information from reviewed case reports and case series of corneal graft rejection.

Source	Age and Sex	Vaccine and Dose	Vaccine Interval Days *	Transplant Interval *	Type of Transplant	Side	Treatment and Outcome
Abousy et al., 2021 [18]	73 F	BNT162b2 #2	4	8 y	DSAEK	OD	Topical prednisolone and hypertonic ointment; resolution
						OS	
Balidis et al., 2021 [19]	77 F	mRNA-1273 #1	7	12 m	DMEK	OS	Topical, subconjunctival, and IV dexamethasone; improvement on the exam, no further follow-up
	64 F	mRNA-1273 #2	7	3 y	PKP	OD	Topical and intracameral dexamethasone; no improvement and additional interventions NR
	69 M	AZD1222 #1	5	22 m	PKP	OD	Subconjunctival and topical dexamethasone, oral methylprednisolone; resolution
	63 M	AZD1222 #1	10	9 m	DSAEK	OS	Topical dexamethasone; no improvement and additional interventions NR
Crnej et al., 2021 [20]	71 M	BNT162b2 #1	7	5 m	DMEK	OD	Topical dexamethasone and oral valacyclovir; resolution
Eduarda Andrade et al., 2022 [21]	40 M	BNT162b2 #1	6	23 y	PKP	OD	Oral prednisone, topical prednisolone, and subconjunctival dexamethasone; resolution
Forshaw et al., 2022 [22]	94 F	BNT162b2 #1	14	24 m	DMEK	OS	Topical dexamethasone, tobramycin, and hypertonic saline; no improvement, repeat DMEK performed in both eyes
				20 m	DMEK	OD	
Marziali et al., 2022 [23]	15 M	BNT162b2 #1	12	18 m	PKP	OD	Topical dexamethasone; resolution
Mohammadzadeh et al., 2022 [24]	36 F	BBIBP-CorV #1	2	4 y	PKP	OS	Topical betamethasone; improvement on the exam, no further follow-up
	54 F	BBIBP-CorV #1	7	2 y	PKP	OS	Topical betamethasone; resolution
Molero-Senosiain et al., 2022 [25]	72 F	BNT162b2 #1	14	4 y	DSAEK	OD	Topical dexamethasone; resolution
	82 F	BNT162b2 #1	14	4 y	DSAEK	OD	Topical steroid (steroid type NR); resolution
	55 M	AZD1222 #1	7	13 y	PKP	OD	Topical steroid (steroid type NR); resolution
	61 M	AZD1222 #2	30	20 y	PKP	OD	Topical steroid (steroid type NR) and IV methylprednisolone; resolution
	48 F	BNT162b2 #1	30	4 y	PKP	OS	Topical dexamethasone; resolution
Nahata et al., 2022 [26]	28 F	AZD1222 #1	14	11 y	FLEK	OS	Topical prednisolone and homatropine and oral methylprednisolone; resolution
Nioi et al., 2021 [27]	44 F	BNT162b2 #1	13	25 y	PKP	OS	Topical dexamethasone and vitamin D supplementation; resolution
Park et al., 2022 [28]	64 M	AZD1222 #1	2	1 y	DSAEK	OS	Topical and oral steroids (steroid types were NR); resolution
Parmar et al., 2021 [29]	35 M	AZD1222 #1	4	6 m	PKP	OS	Topical prednisolone, topical atropine, and IV methylprednisolone; resolution
Phylactou et al., 2021 [30]	66 F	BNT162b2 #1	7	14 d	DMEK	OD	Topical dexamethasone; resolution
	83 F	BNT162b2 #2	21	6 y	DMEK	OD	Topical dexamethasone; improvement of VA and on the exam, no further follow-up
						OS	
Rajagopal and Priyanka, 2022 [31]	79 M	AZD1222 #2	42	4 y	PKP	OS	Topical steroids and oral steroids (steroid types were NR); resolution
Rallis et al., 2022 [32]	68 F	BNT162b2 #1	4	4 m	PKP	OS	Topical dexamethasone and oral acyclovir; resolution
Ravichandran and Natarajan, 2021 [33]	62 M	AZD1222 #1	21	2 y	PKP	OD	NR
Shah et al., 2022 [34]	74 M	mRNA-1273 #1	7	3 m	DMEK	NR	Topical prednisolone; improvement of VA and on the exam, no further follow-up
	61 F	mRNA-1273 #2	7	2.5 y	PKP	NR	Topical prednisolone; improvement of VA and on the exam, no further follow-up
	69 F	mRNA-1273 #2	14	6 y	DSAEK	OS	Topical prednisolone and difluprednate; improvement of VA and on the exam, no further follow-up
	77 M	mRNA-1273 #2	7	22 y	PKP	NR	Topical prednisolone; resolution
Simão et al., 2022 [35]	63 F	CoronaVac #1	1	7 y	PKP	OS	Topical dexamethasone, timolol, bimatoprost, and polydimethylsiloxane; no improvement and additional interventions NR
Wasser et al., 2021 [36]	72 M	BNT162b2 #1	13	1 y	PKP	OS	Topical dexamethasone and oral prednisone; resolution
	56 M	BNT162b2 #1	12	25 y	PKP	OD	Topical dexamethasone and oral prednisone; resolution
Yu et al., 2022 [37]	51 M	mRNA-1273 #1	3	3 w	PKP	OD	Topical steroids (steroid type NR); no improvement and additional interventions NR

* Vaccine interval days = days from reported vaccine to presentation or start of symptoms, transplant interval = time from transplant to vaccination, VA = visual acuity, M = male, F = female, NR = not reported, y = year, m = month, w = week, d = day, OD = right eye, OS = left eye, DSAEK = Descemet’s membrane and automated endothelial keratoplasty, PKP = penetrating keratoplasty, DMEK = Descemet’s membrane endothelial keratoplasty, FLEK = femtosecond laser-assisted endothelial keratoplasty.

**Table 2:** Overview of reported cases of reactivation or recurrence of viral infections and initial herpetic keratitis.

Source	Age and Sex	Vaccine and Dose	Interval Days *	Eye	Diagnosis (Manifestation)	History of Previous Herpetic Keratitis	Treatment
Al-Dwairi et al., 2022 [38]	50 M	BNT162b2 #1	5	OS	HSK (reduced corneal sensation, dendritic ulcers, ciliary injection, AC cells)	Yes	Topical ACV, moxifloxacin, FML, and oral ACV
Alkhalifa et al., 2021 [39]	42 M	NR #1	4	OD	HSK (conjunctival injection, corneal infiltrate with stromal melting, corneal thinning, descemetocele)	Yes	Oral ACV
	29 F	NR #1	28	OS	HSK (epithelial defect and stromal edema, KP)	Yes	Oral ACV, topical ACV, and FML
Alkwikbi et al., 2022 [40]	18 F	BNT162b2 #2	7	OD	HSK (conjunctival hyperemia, dendritic ulcers, KPs, AC cells)	Yes	topical GCV and lubricant drops
	40 M	BNT162b2 #2	7	OD	HSK (dendritic ulcers)	Yes	topical GCV and oral ACV
	32 M	AZD1222 #2	7	OD	HSK (corneal edema, ciliary congestion, dendritic ulcers)	Yes	oral prednisone and topical cyclopentolate
	29 M	BNT162b2 #2	7	OS	HSK (conjunctival injection, stromal infiltration, dendritic ulcers)	Yes	Topical GCV gel and oral ACV
Bolletta et al., 2021 [41]	83 M	BNT162b2 #2	7	OS	Herpetic keratitis, unspecified (manifestation NR)	Yes	Topical ACV
	79 M	AZD1222 #1	5	OD	Herpetic keratitis, unspecified (manifestation NR)	Yes	Oral VCV, topical dexamethasone
	65 F	BNT162b2 #2	6	OS	Herpetic keratitis, unspecified (manifestation NR)	Yes	Oral VCV, topical dexamethasone
Cohen et al., 2022 [42]	81 F	BNT162b2 #2	3	OD	HZO (Vesicular V1 rash, KPs, conjunctival hyperemia)	Yes	Oral VCV, topical dexamethasone, and cyclopentolate
	74 F	BNT162b2 #3	21	OS	HZO (stromal and epithelial edema, KPs)	Yes	Oral VCV and prednisone, topical dexamethasone, and tropicamide
	63 M	BNT162b2 #3	7	OD	HSK (stromal opacity, Descemet folds, KPs, AC inflammation)	No	Oral ACV, topical dexamethasone, and cyclopentolate
Fard et al., 2022 [43]	52 M	BNT162b2 #2	1	OD	Herpetic keratitis, unspecified; likely HSK based on the history of recurrent HSK (stromal haze, punctate epithelial erosions)	Yes	Oral ACV, topical trifluridine, and prednisolone
	67 F	mRNA-1273 #1	NR	OS	Herpetic keratitis, unspecified; likely HZK given the history of HZO (epithelial defect without infiltrate)	Yes	Bandage contact lens, oral VCV, and topical FML
Lazzaro et al., 2022 [44]	46 M	BNT162b2 #1	1	OS	HZO (corneal infiltrates, pseudodendrites, vesicular V1 rash)	Yes	Oral VCV and topical GCV
Li et al., 2021 [45]	60 F	CoronaVac #1	2	OD	HSK (dendritic ulcers)	Yes	Topical GCV
	51 M	CoronaVac #2	NR	OS	HZK (corneal edema, Descemet fold, KP, AC inflammation)	No	oral and topical GCV
Mishra et al., 2021 [46]	71 M	NR #1	10	OD	HZK (panuveitis, KP, AC cells and flare, circumcorneal conjunctival congestion)	No	Oral VCV and steroids, topical steroids, intravitreal GCV
Mohammadpour et al., 2022 [47]	30 F	BBIBP-CorV #	14	OS	Herpetic keratitis, unspecified (central corneal opacity and stromal infiltration)	No	Oral VCV, topical betamethasone
Murgova and Balchev, 2022 [48]	56 M	AZD1222 #2	7	OD	Herpetic keratitis, unspecified (paracentral corneal thinning)	Yes	Topical and oral ACV, oral methylprednisolone
	89 F	BNT162b2 #2	21	OS	Herpetic keratitis, unspecified (ciliary injection, KPs, AC flare)	Yes	Topical methylprednisolone and para-bulbar methylprednisolone injections
Pang et al., 2022 [49]	43 F	NR #2	7	OD	Herpetic keratitis, unspecified (manifestations NR)	NR	Topical GCV, tobramycin, and dexamethasone
	55 F	CoronaVac #2	7	OS	Herpetic keratitis, unspecified; likely HSK based on exam findings (dendritic ulcers)	NR	Topical GCV and cyclosporine
	45 F	NR #1	1	OD	Herpetic keratitis, unspecified; likely HZO based on exam findings (conjunctival congestion, corneal ulcer, vesicular V1 rash)	NR	IV GCV, topical GCV, and cyclosporine
	19 M	NR #2	14	OU	Herpetic keratitis, unspecified (manifestations NR)	NR	IV GCV, topical GCV, and cyclosporine
Rallis et al., 2022 [50]	47 F	AZD1222 #1	4	OS	HSK (anterior uveitis, endotheliitis)	Yes	Oral ACV and topical GCV
	48 M	AZD1222 #1	3	OD	HSK (dendritic ulcers, anterior uveitis)	Yes	Oral ACV and topical GCV
	59 M	AZD1222 #1	4	OU	HSK (OD: dendritic ulcers, OS: geographic ulcer)	No	Oral ACV and topical GCV
	44 M	AZD1222 #1	7	OD	HSK (dendritic ulcers, anterior uveitis)	Yes	Oral ACV and topical GCV
	59 F	AZD1222 #1	5	OD	HZK (pseudodendrites, endotheliitis, anterior uveitis)	Yes	Oral ACV and topical GCV
	65 M	BNT162b2 #1	27	OD	HZO (pseudodendrites, endotheliitis, anterior uveitis, vesicular V1 rash	No	Oral ACV and topical GCV
	95 M	BNT162b2 #1	25	OS	HZO (pseudodendrites, vesicular V1 rash, anterior uveitis)	No	Oral ACV and topical GCV
	89 M	BNT162b2 #1	13	OS	HZO (pseudodendrites, vesicular V1 rash)	No	Oral ACV and topical GCV
	68 M	BNT162b2 #1	28	OD	HZO (pseudodendrites, vesicular V1 rash)	No	Oral ACV and topical GCV
	87 F	BNT162b2 #1	7	OD	HZO (pseudodendrites, vesicular V1 rash)	No	Oral ACV and topical GCV
Rehman et al., 2022 [51]	35 M	AZD1222 #NR	3	OS	HZO (pinpoint fluorescein-positive lesions, circumcorneal congestion, vesicular V1 rash)	No	Oral VCV, topical ACV, moxifloxacin, and carboxymethylcellulose
	40 M	AZD1222 #NR	28	OS	HZO (conjunctival congestion, vesicular V1 rash)	No	Oral VCV and topical moxifloxacin
Richardson-May et al., 2021 [52]	82 M	AZD1222 #1	1	OS	HSK (reduced corneal sensation, dendritic ulcers)	Yes	Oral ACV and doxycycline, topical GCV, prednisolone, atropine, moxifloxacin
Ryu and Kim, 2022 [53]	87 M	BNT162b2 #2	2	OS	HZK (corneal edema, stromal infiltration, corneal neovascularization, KPs)	Yes	Oral VCV and topical prednisolone
Shan et al., 2022 [54]	19 M	CoronaVac #2	21	OU	Suspected herpetic keratitis (conjunctival hyperemia, rough corneal epithelium, patchy corneal infiltration)	NR	IV GCV and topical GCV, and cyclosporine
Song et al., 2021 [55]	30 F	BNT162b2 #1	7	OD	HZK (stromal and endothelial infiltration, corneal edema)	Yes	Topical GCV and loteprednol etabonate
You et al., 2022 [56]	74 M	BNT162b2 #2	5	OS	HZK (conjunctival hyperemia, pseudodendrite) and meningitis	No	IV ACV, topical ACV, and levofloxacin

* Interval days = days from reported vaccination to presentation with symptoms, M = male, F = female, OD = right eye, OS = left eye, OU = both eyes, HSK = herpes simplex keratitis, HZO = herpes zoster ophthalmicus, HZK = herpes zoster keratitis, KP = keratic precipitates, AC = anterior chamber, ACV = acyclovir, VCV = valacyclovir, GCV = ganciclovir, FML = fluorometholone, IV = intravenous.

**Table 3:** Quality scores based on criteria per Task Force for Reporting Adverse Events.

Source	Title	Demographic	Current Health Status	Medical History	Physical Examination	Patient Disposition	Drug Identification	Drug Dosage	Drug Reaction Interface	Concomitant Therapies	Adverse Events	Discussion	Total Points
Abousy et al., 2021 [18]	✓	✓	✓	✓	✓	✓	✓	✓	✓		✓	✓	11
Al-Dwairi et al., 2022 [38]	✓	✓	✓	✓	✓	✓	✓	✓	✓	✓	✓	✓	12
Alkhalifa et al., 2021 [39]	✓	✓	✓	✓	✓	✓	✓	✓	✓	✓	✓	✓	12
Alkwikbi et al., 2022 [40]	✓	✓		✓	✓	✓	✓	✓	✓		✓	✓	10
Balidis et al., 2021 [19]	✓	✓	✓	✓	✓	✓	✓	✓	✓	✓	✓	✓	12
Bolletta et al., 2021 [41]	✓	✓	✓	✓	✓	✓	✓	✓	✓	✓	✓	✓	12
Cohen et al., 2022 [42]	✓	✓	✓	✓	✓	✓	✓	✓	✓	✓	✓	✓	12
Crnej et al., 2021 [20]	✓	✓	✓	✓	✓	✓	✓	✓	✓		✓	✓	11
de la Presa et al., 2022 [57]	✓	✓	✓	✓	✓	✓	✓	✓	✓	✓	✓	✓	12
Eduarda Andrade et al., 2022 [21]	✓	✓	✓	✓	✓	✓	✓	✓	✓	✓	✓	✓	12
Fard et al., 2022 [43]	✓	✓	✓	✓	✓	✓	✓	✓	✓	✓	✓	✓	12
Farrell et al., 2022 [58]	✓	✓	✓	✓	✓	✓	✓	✓	✓	✓	✓	✓	12
Forshaw et al., 2022 [22]	✓	✓	✓	✓	✓	✓	✓	✓	✓	✓	✓	✓	12
Gouveau et al., 2022 [59]	✓	✓	✓	✓	✓	✓	✓	✓	✓	✓	✓	✓	12
Khan et al., 2021 [60]	✓	✓	✓	✓	✓	✓	✓	✓	✓	✓	✓	✓	12
Lazzaro et al., 2022 [44]	✓	✓		✓	✓	✓	✓	✓	✓		✓	✓	10
Lee and Han, 2022 [61]	✓	✓	✓	✓	✓	✓	✓	✓	✓	✓	✓	✓	12
Li et al., 2021 [45]	✓	✓	✓	✓	✓	✓	✓	✓	✓	✓	✓	✓	12
Marziali et al., 2022 [23]	✓	✓	✓	✓	✓	✓	✓	✓	✓	✓	✓	✓	12
Mishra et al., 2021 [46]	✓	✓	✓	✓	✓	✓	✓	✓	✓		✓	✓	11
Mohammadopur et al., 2022 [47]	✓	✓	✓	✓	✓	✓	✓		✓	✓	✓	✓	11
Mohammadzadeh et al., 2022 [24]	✓	✓	✓	✓	✓	✓	✓	✓	✓	✓	✓	✓	12
Molero-Senosiain et al., 2022 [25]	✓	✓	✓	✓	✓	✓	✓	✓	✓	✓	✓	✓	12
Murgova and Balchev, 2022 [48]	✓	✓		✓	✓	✓	✓	✓	✓		✓	✓	10
Nahata et al., 2022 [26]	✓	✓	✓	✓	✓	✓	✓	✓	✓	✓	✓	✓	12
Nioi et al., 2021 [27]	✓	✓	✓	✓	✓	✓	✓	✓	✓		✓	✓	11
Pang et al., 2022 [49]	✓	✓		✓	✓	✓		✓	✓		✓	✓	9
Park et al., 2022 [28]	✓	✓	✓	✓	✓	✓	✓	✓	✓		✓	✓	11
Parmar et al., 2021 [29]	✓	✓	✓	✓	✓	✓	✓	✓	✓	✓	✓	✓	12
Penbe, 2022 [62]	✓	✓		✓	✓	✓	✓	✓	✓	✓	✓	✓	11
Phylactou et al., 2021 [30]	✓	✓	✓	✓	✓	✓	✓	✓	✓	✓	✓	✓	12
Rajagopal and Priyanka 2022 [31]	✓	✓	✓	✓	✓	✓	✓	✓	✓		✓	✓	11
Rallis et al., 2021 [32]	✓	✓	✓	✓	✓	✓	✓	✓	✓	✓	✓	✓	12
Rallis et al., 2022 [50]	✓	✓	✓	✓	✓	✓	✓	✓	✓	✓	✓	✓	12
Ravichandran and Natarajan 2021 [33]	✓	✓	✓	✓	✓		✓	✓	✓	✓	✓	✓	11
Rehman et al., 2022 [51]	✓	✓	✓	✓	✓	✓	✓		✓	✓	✓	✓	11
Richardson-May et al., 2021 [52]	✓	✓		✓	✓	✓	✓	✓	✓		✓	✓	10
Ryu and Kim, 2022 [53]	✓	✓	✓	✓	✓	✓	✓	✓	✓		✓	✓	11
Shah et al., 2022 [34]	✓	✓	✓	✓	✓	✓	✓	✓	✓	✓	✓	✓	12
Shan et al., 2022 [54]	✓	✓		✓	✓	✓	✓	✓	✓		✓	✓	10
Simao et al., 2022 [35]	✓	✓	✓	✓	✓	✓	✓	✓	✓	✓	✓	✓	12
Song et al., 2021 [55]	✓	✓	✓	✓	✓	✓	✓	✓	✓		✓	✓	11
Wasser et al., 2021 [36]	✓	✓	✓	✓	✓	✓	✓	✓	✓	✓	✓	✓	12
You et al., 2022 [56]	✓	✓	✓	✓	✓	✓	✓	✓	✓		✓	✓	11
Yu et al., 2022 [37]	✓	✓	✓	✓	✓	✓	✓	✓	✓		✓	✓	11

## Data Availability

Not applicable.
